# Effect of Supplemental Light for Leaves Development and Seed Oil Content in *Brassica napus*

**DOI:** 10.3390/genes15111371

**Published:** 2024-10-24

**Authors:** Xingying Yan, Wenqin Bai, Taocui Huang

**Affiliations:** 1 College of Agronomy and Biotechnology, Southwest University, No. 2 Tiansheng Road, Beibei, Chongqing 400716, China; yxingying@swu.edu.cn; 2 Chongqing Academy of Agricultural Sciences, Nongke Road, Jiulongpo, Chongqing 401329, China; bwqbc82@126.com

**Keywords:** *Brassica napus*, light intensity regulation, leaf phenotypes, seed oil content, transcriptome

## Abstract

Rapeseed is an important commercial crop globally, used for both animal fodder and human consumption. Varied insolation duration and intensity are among the main factors affecting the seed yield and quality of *Brassica napus* (*B. napus*) worldwide. In this study, the high-oil-content rapeseed cultivar “Qingyou 3” was subjected to a light supplementation trial during both the vegetative growth period and the seed productive stage. Different light intensity conditions were stimulated using light-emitting diodes (LEDs). The main plot factor was land condition, with LED treatment (Treatment) and without LED treatment (Control) under natural conditions. The results showed that the leaf size and thickness, photosynthesis efficiency, and seed oil content of *B. napus* increased significantly after light supplementation. Then, 18 cDNA libraries were constructed from leaf segments (30 days after transplanting—DAT) and seeds 30 and 40 days after pollination (DPA) for RNA transcriptome sequencing. It was found that genes encoding lipid transfer protein, phenylpropanoid biosynthesis, photosynthesis, and plant hormone signal transduction were enriched in differentially expressed genes (DEGs). The qRT-PCR analysis showed that eight key genes had significant variations, a finding also consistent with the RNA-seq results. The aim of this study was to identify the DEGs and signaling pathways in the leaves and seeds of *B. napus* during the vegetative and seed productive stages under different light intensities. The results provide insight into how sufficient light plays a critical role in promoting photosynthesis and serves as the foundation for material accumulation and yield formation.

## 1. Introduction

Sunlight is the most prominent factor for plant development, varying dynamically across land surfaces and ranging from milliseconds to hours [1,2]. Sunlight intensity depends on the instantaneous amount of light, as well as the cumulative light delivered each day. In addition to geographic location and environmental factors, plants also have to face insufficient light due to global climate change. Changes in light density or duration for crops are usually caused by variations in solar irradiance and shading from the overlapping leaves of surrounding plants [3]. In recent years, solar irradiance has decreased by 28–49% because of heavy aerosol and haze pollution induced by the development of industry and vehicle exhaust emissions [4]. Insufficient light has become an ongoing global phenomenon and will significantly impact crop production [5]. Thus, it is important to research the effects of light supplementation on plant breeding.

Rapeseed (*B*. *napus* L.) is one of the most important sources of protein and oil produced worldwide [6]. The southwest of China is a representative rapeseed production region; however, rainy and cloudy conditions often occur in Sichuan and Chongqing during the developmental stage of rapeseed (October to the following March), leading to low light intensity that severely negatively impacts rapeseed quality and yield. In contrast, Yunnan province experiences much higher light intensity and duration, resulting in higher seed oil content and yield of rapeseed compared to other districts.

A previous study found that supplementary far-red (FR) light (lower red (R)/FR conditions) can improve leaf area and light absorption in *Arabidopsis thaliana* (*A. thaliana*) [7], leading to higher biomass and stronger resistance to low temperature compared to those grown under higher R/FR conditions [8]. The electron transport rate, photochemical efficiency, and net photosynthetic rate all affect the capacity for carbon acquisition and assimilation. Additionally, reduced sunlight leads to a decrease in the chlorophyll a/b ratio and key enzyme activities in photosynthesis (e.g., phosphoenolpyruvate carboxylase) [9,10,11]. Therefore, measuring photosynthetic indicators would help us understand effective light utilization in crops, especially in greenhouse farming.

The light utilization mechanism in plants, including *B. napus*, is complex, with lighting parameters (e.g., photoperiod, intensity, and quality) varying significantly. Unstable light environments may negatively impact plant cultivation, especially during winter and rainy periods when natural light is often insufficient to support maximum growth. Since light intensity changes over the seasons on different timescales, the addition of light-emitting diodes (LEDs) is an effective way to offset insufficient lighting. LEDs are often used in indoor farming to precisely control light intensity, allowing for controlled conditions such as light spectral composition, location, and timing [12]. Among visible light, red-orange light (600~700 nm), and blue-violet light (400~500 nm) are the major absorbed elements, while only a small amount of green light (500~600 nm) can be absorbed by green plants [13]. The combined spectrum of red and blue light can improve the dry matter quality, leaf number, and seed yield in wheat [14] and can also improve the dry matter quality of lettuce [15]. Previous studies have shown that supplemental LEDs can effectively regulate crop growth and accelerate maturity by enhancing light and CO_2_ absorption and assimilation through increased photosynthesis in tomato plants [16,17]. Similarly, a 3.5–5.7-fold increase in the leaf photosynthetic rate of inner canopy foliage and a 30% yield increase were observed when intracanopy LED lighting was applied to pepper plants [18]. Therefore, a lower R:FR condition of LEDs was selected in this research to examine the development of *B. napus*.

The seed oil content of rapeseed is generally between 35% and 50%, which is mainly determined by genetic effects and genotype × environment interactions (GE). In recent years, transcriptome sequencing has been widely used to investigate the formation mechanism of seed oil content. Rapeseed seeds from four varieties were analyzed based on transcriptome sequencing, revealing that the genes involved in lipid synthesis are conserved and species-specific [19]. The three different parts of oil palm fruits and seeds were analyzed through transcriptomics, revealing that EgWRI1-1 and EgWRI1-2 transcription factors were heavily transcribed in the process of oil accumulation in the pericarp and endosperm [20]. The expression of lipid metabolites and oil-synthesis-related genes were compared between high- and low-oil-content varieties, and the results showed significant differences in different parts of the seeds [21]. Thus, some key regulatory genes related to oil synthesis can be identified through transcriptome analysis of differentially expressed genes (DEGs) in rapeseed seeds, providing a theoretical basis for future high-oil rapeseed breeding.

However, to date, few studies have focused on the effect of light intensity on plants throughout their entire life cycle. Therefore, a light intensity control experiment (rapeseed grown under natural conditions and light supplementation by LED) was conducted in Chongqing, China, for this study. The main objectives of this study were to investigate the DEGs and phenotypes of leaf growth and seed oil content regulated by different light intensity in rapeseed. These findings can provide information on efficiently utilizing light for rapeseed breeding in regions affected by poor solar irradiance.

## 2. Method

### 2.1. Plant Materials and Experimental Design

**Figure 1:**
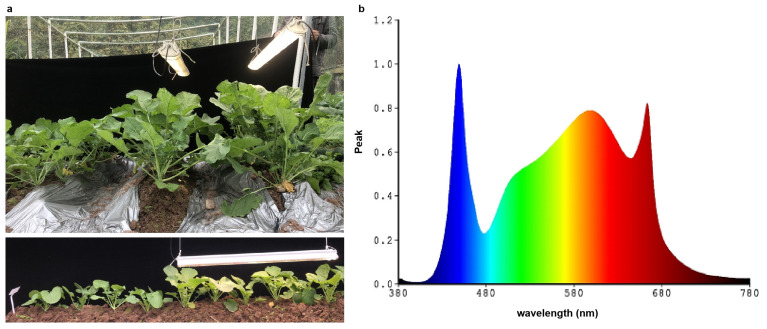
Different light intensity conditions were provided by LEDs in the field. (**a**) Comparison of plants supplied light directly to the leaves by LEDs from 7:00 AM to 19:00 PM versus control; (**b**) Wavelengths of the LEDs used in the trial.

The *B*. *napus* variety Qingyou 3 was used in this experiment as it is broadly adaptable in China. The variety Qingyou 3 also has a high seed oil content and yield. Field experiments were conducted in Beibei District (29°48′ N, 106°24′ E), Chongqing, China between September 2021 and May 2022. Seedlings were raised in a seedbed for 30 days, and then uniform healthy plants were transplanted into individual pots spaced 50.0 × 20.0 cm apart. There were 30 pots for each treatment, resulting in a total of 60 pots. Half a month after transplanting, the temperature in early winter was approximately 10–15 °C. Compared to natural conditions as a control, light supplementation treatment was applied to the test groups. Light supplementation was provided using LEDs (KS-Z-D60, China) (Figure 1a), and the wavelength of the LEDs is shown in Figure 1b. The light supplementation time was from 7:00 am to 19:00 pm. A spectrometer (HR-550, Taiwan Hipoint Corporation, Taiwan, China) was used to measure the luminance.

After 30 days of light treatment, when the plants had grown 10 true leaves, the second unfolded leaf from the top of each representative individual plant was collected. Leaf samples were immediately frozen in liquid nitrogen and stored at −80 °C for transcriptome sequencing and hormone (ABA and IAA) analysis. Free IAA and ABA measurements were analyzed using liquid chromatography–tandem mass spectrometry as described previously [22]. Seeds from 30 and 40 days after pollination (DAP) were also collected for RNA extraction, followed by transcriptome sequencing. Thus, 6 samples with 3 biological replicates each were collected for transcriptome sequencing.

At harvest, 10 representative individual plants from each treatment group were selected. The seed oil content and protein content were determined using a near-infrared rapid quality analyzer (NIR) (Foss NIR Systems 6500, Hillerød, Denmark) according to the method outlined by Kaur et al. (2016) [23].

### 2.2. Microscopic Observations

Three middle segments of leaves (1 cm^2^) were sampled for paraffin sectioning. The segments were fixed in FAA (10% formaldehyde, 5% acetic acid, and 50% ethanol) at 4 °C under vacuum for 2 h, dehydrated through a graded ethanol series, cleared in dimethylbenzene, and embedded in paraffin as described previously [24]. Cross-sections were cut using a RM2125 microtome (Leica, Wetzlar, Germany) and stained in toluidine blue. Leaf sections from different light conditions were imaged using an BX53 microscope (Olympus, Tokyo, Japan). The thickness of the leaves, including spongy tissue and palisade tissue in each segment, was measured using Image J (https://imagej.net/contribute/citing).

### 2.3. Photosynthetic Performance Test

The rapeseed leaves were used to measure net photosynthetic efficiency, stomatal conductance, and intercellular CO_2_ concentration using an Li-6400 (Li-Cor, Lincoln, NE, USA) 30 days after treatment and before the flowering stage. Five fully expanded leaves were selected with a southeast orientation, consistent growth and representative characteristics as materials from 9:00 to 11:00 a.m. at 12 °C. The net photosynthetic rate (Pn) and related parameters were measured in the middle of fixed leaves using the light source of the instrument. The CO_2_ concentration in the air was adjusted to 380 μmol·mol^−1^, and the gas source was air two meters away from the experimental area. The measurement method followed the instructions for use, with 10 data points recorded each time. The mean value was used for data analysis. 

### 2.4. Differential Expression Analysis and Functional Annotation

The reference genome used was obtained from *B. napus* genome (version 5.0; http://www.genoscope.cns.fr/brassicanapus/). Samples were collected in 3 replicates from rapeseed leaves and seeds at 30 and 40 DPA. RNA from all these samples was subjected to transcriptome sequencing by Majorbio Technologies Co., Ltd. (Shanghai, China). Gene expression levels were measured in terms of reads per kilobase of transcript per million mapped reads (RPKM). DEGs were defined with a false discovery rate (FDR) of <0.01 and |log2(fold change)|≥1. Two samples were compared using the “LEDs vs. Control” method. If the expression level of a DEG in the “LEDs” sample was higher than that in the “Control”, DEG was considered to be upregulated; otherwise, it was classified as downregulated. To further characterize the functions of DEGs, they were mapped to Gene Ontology (GO) classifications using Blast2GO [25], and Kyoto Encyclopedia of Genes and Genomes (KEGG) enrichment was analyzed using KOBAS [26].The data were analyzed online on the Majorbio Cloud Platform.

### 2.5. qRT-PCR Analysis and Statistical Analysis

The primers were designed using PRIMER-BLAST of NCBI’s primer designing tool (Table S1). The Bio-Rod quantitative PCR instrument was adopted to perform real-time fluorescence quantitative PCR. The following thermocycle conditions were used: 95 °C for 5 min, 38 cycles at 95 °C for 15 s, 56 °C for 15 s, and 72 °C for 15 s, respectively. The *B. napus* actin gene (AF1l1812) was selected as a reference gene in this study. All data were obtained from three biological repetitions, and experiments were repeated three times. Student’s *t*-tests were used to demonstrate significant differences (*, *p* < 0.05; **, *p* < 0.01) between the control group and the treatment group.

## 3. Results and Discussion

### 3.1. Light Supplementation Has an Effect on Leaf Development and the Seed Oil Composition of B. napus Plants

**Figure 2:**
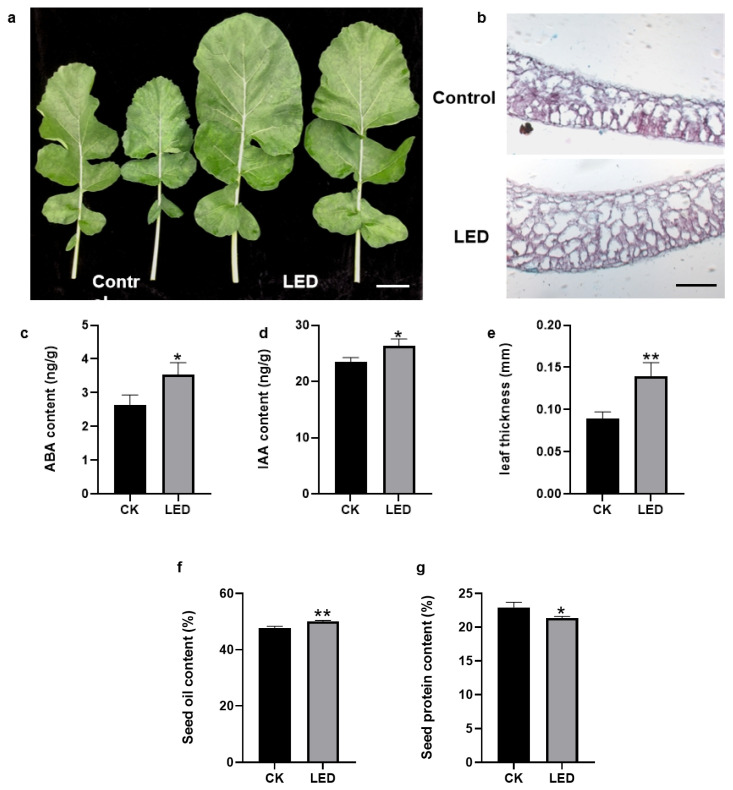
Phenotypic observations of leaves between the control and LED treatment groups. (**a**) Comparison of leaf size between the control and LED treatment groups, bars = 5 cm; (**b**) Histological analysis of the leaf blades. The difference in leaf thickness between the control and LED treatment groups assessed through microscopic observations, bars = 100 μm; (**c**) The ABA content of leaves under the control and LED treatment; (**d**) The IAA content of leaves under the control and LED treatment; (**e**) Comparison of leaf thickness through microscopic observations between the control and LED treatment groups; (**f**) Seed oil contents of control and LED treatment groups; (**g**) Seed protein contents of control and LED treatment groups (** *p* < 0.01 and * *p* < 0.05)**.** CK, Control treatment; LED, LED treatment.

As shown in Figure 1b, the LEDs are enriched at wavelengths of 420–475 nm and 500–650 nm. Although the irradiation was variable under natural conditions, the light intensity is relatively fixed at 8093.3 lux under the LEDs (Figure 1a). To explore the change in leaf growth induced by light intensity supplementation, the leaf size, leaf anatomical structure, hormone concentration, and photosynthetic efficiency in rapeseed leaves were investigated. The size of leaves under LEDs was significantly larger than that of corresponding leaves under control conditions (Figure 2a), which is consistent with the results reported by [11]. The thickness of leaves under LEDs increased by 50% compared to the control, as observed in cross-section microscopic images (Figure 2b,c). A previous study found that shade affects cell proliferation and hormones, which may play significant roles in determining leaf size [27]. The results implied that these hormones may boost the mechanisms of growth and developmental processes in the plants under LEDs, as the *YABBY* genes play significant roles in leaf lamina development in *A*. *thaliana* [28], snapdragon (*Antirrhinum majus*) [29], and rice (*Oryza sativa*) [30]. Additionally, it has been found that *OsIAA1* exhibits different expression patterns under light and dark conditions, indicating that certain *Aux/IAA* genes are also involved in the light signaling pathway [31]. Net photosynthetic efficiency, stomatal conductance, and intercellular CO_2_ concentration were also significantly increased in the plants under LEDs (Table 1). Additionally, the concentrations of ABA and IAA significantly increased in the plants under LEDs (Figure 2d,e). These results indicate that the intensity of light reaching the leaves influences the growth process of leaves. We also measured the seed oil content and protein content of mature seeds using NIR. After light supplementation, the seed oil content reached up to 50%, significantly higher than in the control, although the protein content decreased slightly (Figure 2f,g).

### 3.2. Overview of Transcriptome Analysis and DEGs

**Figure 3:**
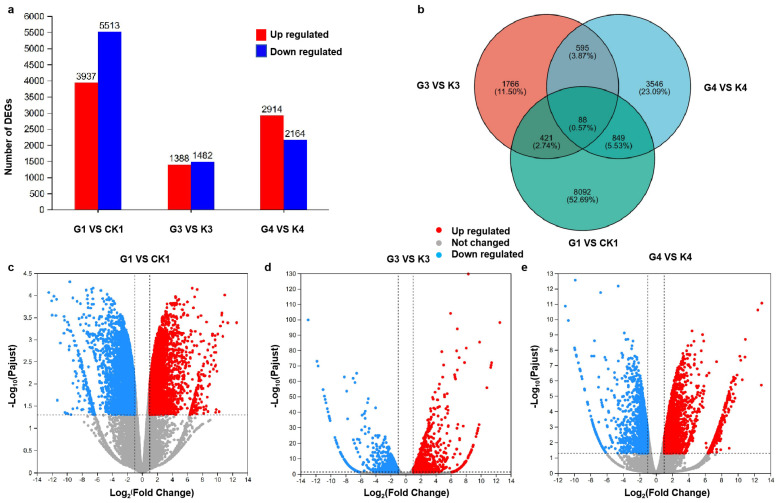
(DEGs: (**a**) number of DEGs in each group; (**b**) Venn diagrams for six groups; (**c**) volcano plot for G1 vs. K1; (**d**) volcano plot for G3 vs. K3; (**e**) volcano plot for G4 vs. K4.

A total of 18 samples from 6 groups, including K1 (leaves under control), G1 (leaves under LED), K30 (30 DPA seed of control), G30 (30 DPA seed under LED), K40 (40 DPA seed of control), and G40 (40 DPA seed under LED), were subjected to high-throughput Illumina sequencing to obtain an overview of the gene expression profiles of *B. napus* leaves and seeds in response to changes in light intensity. DEGs analysis was performed through pairwise comparison in three comparison groups (G1 vs. K1, G3 vs. K3, and G4 vs. K4), generating a total of 17398 DEGs, among which 9815 (5513, 1388, and 2914, respectively) were upregulated and 7583 (3937, 1482, and 2164, respectively) were downregulated (Figure 3a). The expression levels of up- and downregulated genes in different comparison groups were visualized using volcano plots (>2-fold change, false discovery rate < 0.05) (Figure 3c–e). The color differences indicate upregulation (red color) and downregulation (green color). A Venn diagram was used to visualize the common DEGs in three comparison groups, showing that 88 common DEGs exist in all comparison groups, while 683 DEGs were commonly expressed in G3 vs. K3 and G4 vs. K4 (Figure 3b).

### 3.3. Enrichment Characteristics of KEGG and GO of DEGs

**Figure 4:**
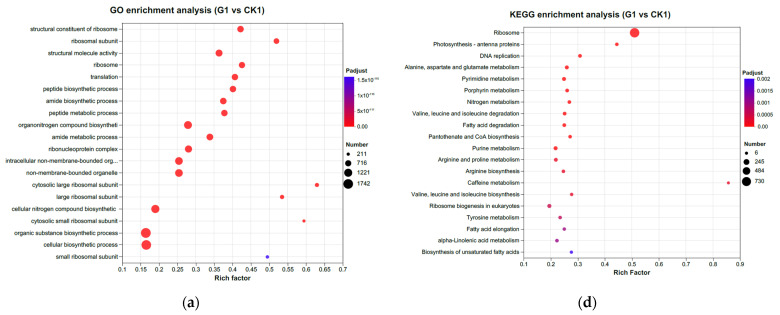

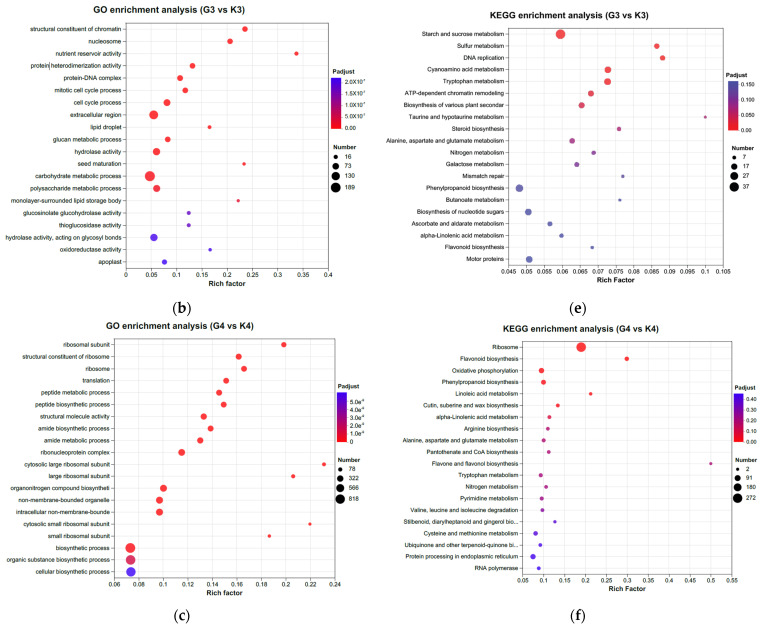
GO and KEGG analysis of DEGs between control and LED treatment. The rich factor is the ratio of the number of DEGs to the total number of genes with a specific function. The color and size of the dots represent the range of the padjust < 0.5 and the number of DEGs mapped to the indicated function, respectively. (**a**) GO analysis of DEGs in the leaves; (**b**) GO analysis of DEGs in the 30 DPA seeds; (**c**) GO analysis of DEGs in the 40 DPA seeds; (**d**) KEGG analysis of DEGs in the leaves; (**e**) KEGG analysis of DEGs in the 30 DPA seeds; (**f**) KEGG analysis of DEGs in the 40 DPA seeds.

In terms of GO classification, the DEGs in G1 vs. K1 are primarily assigned to the categories of “cellular biosynthetic process”, “organic substance biosynthetic process”, and “organonitrogen compound biosynthetic process” (Figure 4a). The most enriched GO categories in G3 vs. K3 were “carbohydrate metabolic process”, “extracellular region”, “polysaccharide metabolic process”, “cell cycle process”, and so on (Figure 4b). The GO terms “biosynthetic process”, “organic substance biosynthetic process”, and “cellular biosynthetic process” were commonly enriched in G4 vs. K4 (Figure 4c). KEGG enrichment analysis of G1 vs. K1 showed that the most significant DEGs were enriched in plant hormone signal transduction and phenylpropanoid biosynthesis (Figure 4d). After treatment with LEDs, the DEGs for 30 DPA seeds were mainly enriched in starch and sucrose metabolism (Figure 4e). After treatment with LEDs, for 40 DPA seeds, the most enriched pathways in G4 vs. K4 were related to phenylpropanoid biosynthesis, excluding ribosome-related genes (Figure 4f). Photosynthesis and photosystem Ⅱ assembly are among the enriched biological pathways identified by KEGG analysis. In our study, among the DEGs related to the photosynthetic system, the expression of BnaCnng55860D, BnaA09g26570D, BnaC02g44590D, BnaA07g07570D, BnaC08g38660D, and BnaAnng00160D was significantly increased (Supplementary Table S1). Thus, the present study found that these genes serve as positive regulators that indirectly promote leaf development. In addition, a group of xyloglucan endotransglucosylase/hydrolase (XTH) proteins was constantly upregulated in leaf tissues, with XTH22 showing high expression in response to light supplementation compared to control conditions. This protein is associated with the elongation of cell walls [32].

### 3.4. Light Supplementation Induced DEGs in Leaves

In terms of hormonal regulation pathways, the DEGs of leaves after treatment with LEDs included Auxin efflux carrier, Aux/IAA, and SAUR. These essential genes respond to auxin induction within a very short timeframe and are classified as early auxin response genes [33]. The expressions of Auxin efflux carrier (BnaA09g09160D), Aux/IAA (BnaC06g40600D), and SAUR (BnaA09g56290D) exhibited significant upregulation, with increases of 8.6-fold, 9.4-fold, and 6.6-fold, respectively. The expressions of PYL6 (BnaA03g19030D) and PYL6 (BnaA07g38130D) were upregulated 4.0- and 4.6-fold, respectively. The expression levels of abscisic acid 8′-hydroxylase (ABAH), which are crucial genes in the ABA metabolism pathway, were found to be upregulated. As expected, we identified five upregulated DEGs coding for chlorophyll A-B binding protein processes. It has excellent blue–green light capture capability and strong light protection, providing the energy basis for the rapid growth and reproduction of diatom cells [34]. The GRAS gene family is a kind of transcription factor that is widely distributed in plants, playing a significant role in plant growth and development, biological and abiotic stress, light signaling, hormone signal responses, and other processes. Some GRAS transcription factor members were upregulated, which may play a vital role in leaf growth and development. We also screened other transcription factors (TFs) with differential expression profiles belonging to important families involved in plant development pathways, such as MYB, MADS, and TCP, which exhibited an increasing trend in expression following LED treatment. Furthermore, the key genes in cell division control proteins exhibited distinct expression patterns between the control and LED treatments (Figure 4 and Supplementary Figure S1). DEGs were highly clustered in flavonoid and phenylpropanoid biosynthesis processes, suggesting that these genes may function through these pathways. The “flavonoid” was identified as the one of the most significantly enriched pathways in KEGG analysis (rich factor = 0.23 and Q value < 0.001). This significance may be attributed to the relationship between oil storage changes and the color change in seed coatings during seed maturation.

### 3.5. Candidate Genes Involved in Seed Oil Content Synthesis

Notable changes were observed in the genes involved in seed storage protein, plant lipid transfer proteins, late embryogenesis abundant (LEA) proteins, and SWEET sugar transporters in the seed profile. In terms of plant lipid transfer proteins, the expression of BnaC01g19330D exhibited a significant 2-fold upregulation in 30 DPA seeds under LEDs and 1.75-fold upregulation in 40 DPA seeds under LEDs. The expression levels of BnaA01g17200D exhibited a 3.9-fold increase in 30 DPA seeds and a 1.5-fold increase in 40 DPA seeds under LEDs. BnaC03g08840D exhibited a remarkable 3.4-fold upregulation under LEDs of 40 DPA seeds; however, its expression increased by only 1.5-fold in 30 DPA seeds under the same conditions. Furthermore, the expression levels of BnaAnng06900D increased by 2.9-fold in 40 DPA seeds and 1.4-fold in 30 DPA seeds. Regarding LEA proteins, LEA-3 and LEA-25 were most significantly upregulated in 30 DPA seeds under LEDs but downregulated in 40 DPA seeds under LEDs. In contrast, SWEET sugar transporters were most significantly downregulated in 30 DPA seeds under LEDs but upregulated in 40 DPA seeds under LEDs. A large proportion of TFs are upregulated in G4 vs. K4, indicating that most gene expressions are stimulated under LEDs during the late stages of seed development. Furthermore, the key genes in the bidirectional sugar transporter SWEET (i.e., SWEET1) exhibited distinct expression patterns between the control and light treatments. In densely planted crops, the oil composition in different light environments may be changed by low R:FR ratios due to mutual shading [35]. In this study, sugar transport was shown to play a crucial role in oil content biosynthesis and metabolism.

### 3.6. qRT-PCR Analysis

**Figure 5:**
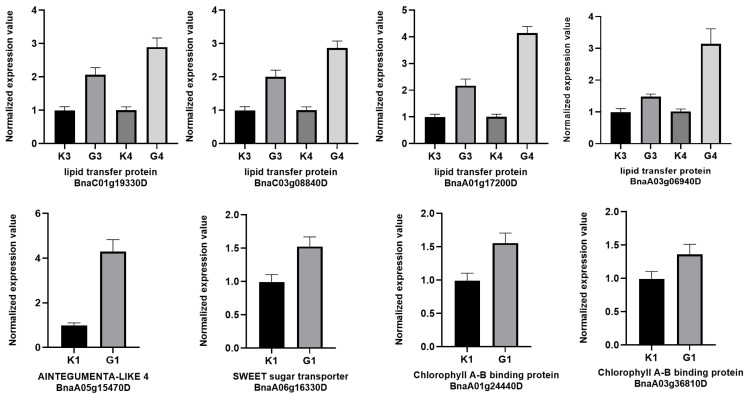
qRT-PCR analysis of DEGs related to leaf and seed development in the control and LED treatment groups.

Real-time quantitative PCR analysis was performed to validate the expression of eight randomly selected genes in samples under LEDs and their respective controls. The qRT-PCR results were found to be consistent with the RNA-seq data (Figure 5).

Taken together, these results indicate that light supplementation increases leaf size through enhanced photosynthesis efficiency, cell proliferation, and hormone content. This suggests that LEDs are useful for increasing biomass in plant production. These findings align with analyses of the model plant species *A*. *thaliana*, which show that the plant’s response to shade is due to the regulation of changes in light intensity and quality by integrating light and auxin signals [36]. There were some candidate gene were found to response LEDs treatment, but there function is still needed to study. 

## 4. Conclusions

The results of this study comparing *B. napus* under natural conditions with those light supplemented by LEDs explain the reason why the seed oil content is higher in some regions with sufficient solar resources. The leaf development and seed oil content in *B. napus* increased with higher light intensity. These results significantly contribute to light utilization in crop breeding, aided by high-throughput technology. They also highlight the fact that the seed oil content is not controlled by a single gene but involves the coordinated expression of multiple genes, elucidating the underlying mechanisms of light-regulated growth in *B. napus* through a light-mediated transcriptional regulation network. The identification of these genes opens new avenues for light utilization in crop breeding and has implications for the development of cultivars with higher photosynthetic efficiency through transgenic technology. In conclusion, this study emphasizes the importance of understanding the complex processes involved in plant light utilization.

## Figures and Tables

**Table 1:** Photosynthetic indices in the control and the LED treatment groups.

Lines	Photosynthetic Rate	Intercellular CO_2_ Concentration	Stomatal Conductance
Control	5.28 ± 007	79.1571 ± 4.8	0.36 ± 0.09
LEDs	5.88 ± 0.13 **	86.6714 ± 0.62 **	0.55 ± 0.04 **

Note: values are means±standard error (n = 5), **: significantly different at the *p* < 0.01 level.

## Data Availability

The datasets generated during and/or analyzed during the current study are available from the corresponding author on reasonable request.

## Supplementary Materials

The following supporting information can be downloaded at: https://www.mdpi.com/article/10.3390/genes15111371/s1, Figure S1: Expression profile of pathway. (a) Phenylpropanoid biosynthesis; (b) Stach and sucrose metablism; (c) Flavonoid biosynthesis; (d) Indole-3-acetic acid (IAA) signaling pathway; (e) abscisic acid (ABA) signaling pathway. Solid lines and arrows indicate the signal transduction process and direction. Red frame means up-regulation, Blue frame means down-regulation, Yellow means known genes, Green means new genes; Table S1: Genes and primers used in qRT-PCR.
