# Supplementary material for: Effect of Supplemental Light for Leaves Development and Seed Oil Content in Brassica napus
Source: Genes (Basel). 2024 Oct 24;15(11):1371. doi: 10.3390/genes15111371 (PMC11594260; doi:10.3390/genes15111371)
Supplement: Supplementary file 1 [file genes-15-01371-s001.zip › genes-3199847-supplementary.pdf]

Table S1 Genes and primers used in qRT-PCR.

Gene ID	Forword Primers	Reverse Primers	Description
BnaC01g19330D	ACAGGACGGTTGTGGAAGTC	CAGACTGCATTGCCTGCTTG	Lipid transfer protein
BnaC03g08840D	CCAATGCCTCCGTCTACAGG	CCTAAATGGACCGGCTTGGT	Lipid transfer protein
BnaA01g17200D	AGCCGGCCCATTTAGGATTC	ACTGCATTGCTTGCTTGTGG	Lipid transfer protein
BnaA03g06940D	ACAGGACGGTTGTGGAAGTC	GCTTGTGGAGCCATTGTTGG	Lipid transfer protein
BnaA05g15470D	TTTCAATGGGAAGTGCCGGA	TCTAGGTGCAGCTTCAACGG	Protein AINTEGUMENTA-LIKE 4
BnaA06g16330D	CTCTCCTGTGCCAACGTTCT	TGGTGACGAGGAGGAAGACA	SWEET sugar transporter
BnaC08g20440D	AAGTTACCGGAGGTGACCCT	ACGAACGGCTGTGATTGTCT	Chlorophyll A-B binding protein
BnaA03g36810D	CACCATCGGAGATCTGAGCC	CCTTGAAAAGGGGCACAAGC	Chlorophyll A-B binding protein

a

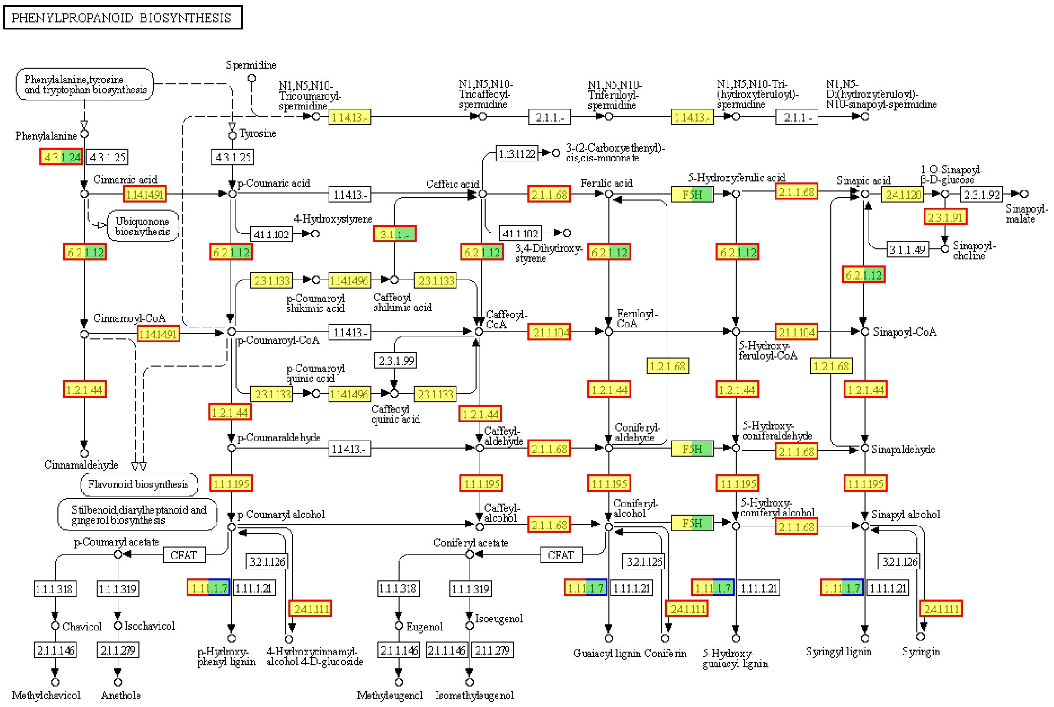

b

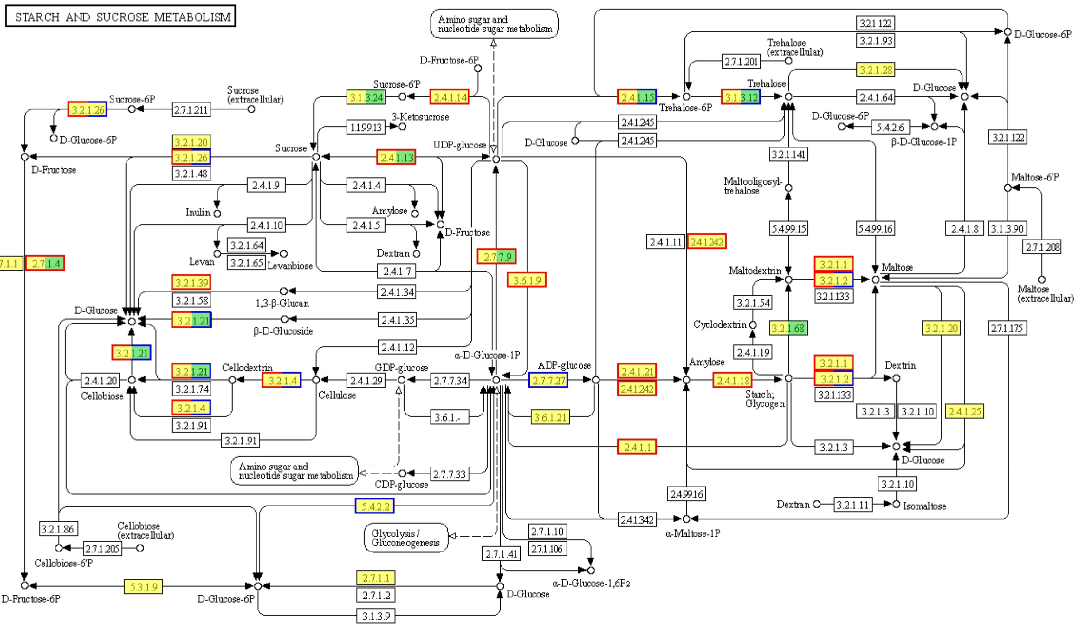

c

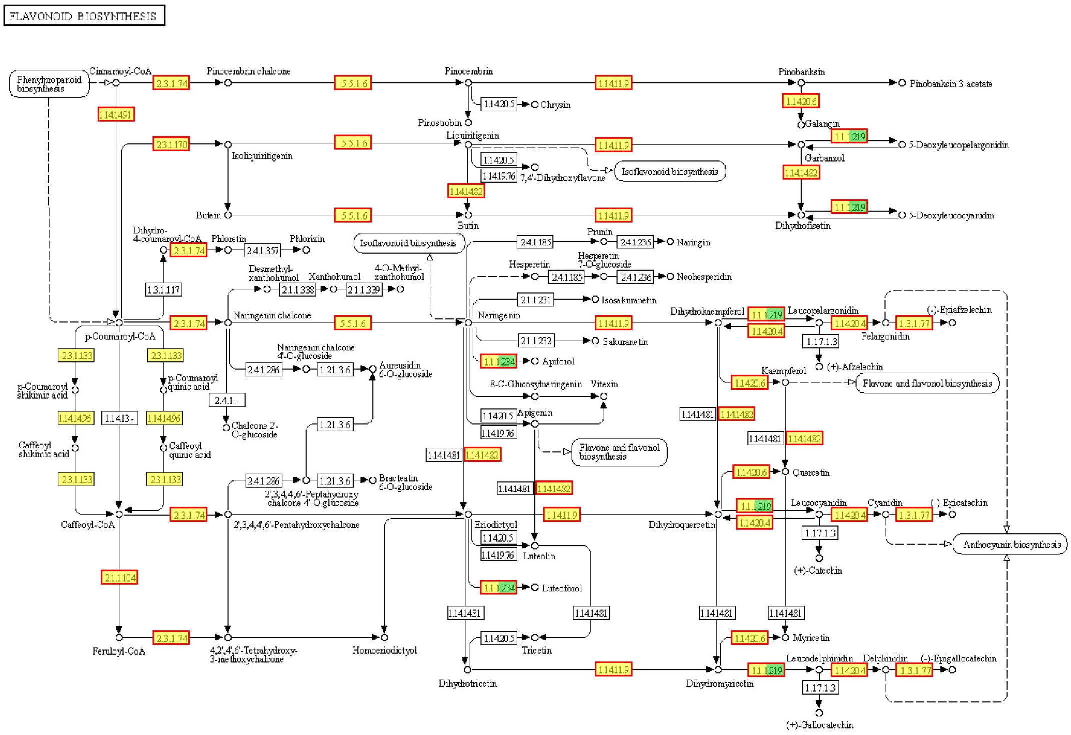

d

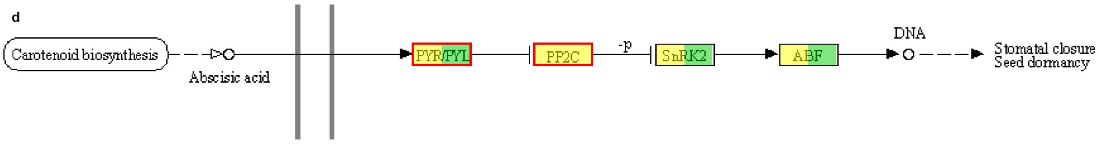

e

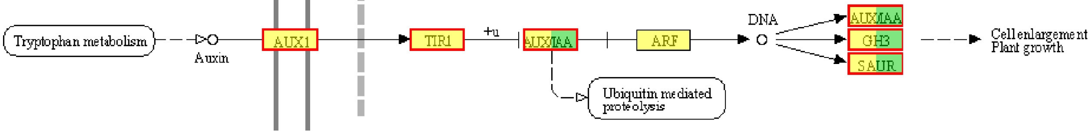

Supplementary Figure S1 Expression profile of pathway. (a) Phenylpropanoid biosynthesis; (b) Stach and sucrose metablism; (c) Flavonoid biosynthesis; (d) Indole-3-acetic acid (IAA) signaling pathway; (e) abscisic acid (ABA) signaling pathway. Solid lines and arrows indicate the signal transduction process and direction.

Red frame means up-regulation, Blue frame means down-regulation, Yellow means known genes, Green means new genes.
